# Seasonal Habitat Distribution and Connectivity Response of Water Deer and Wild Boar to Hotspot Fencing in a Fragmented Urban Forest Fringe

**DOI:** 10.1002/ece3.73000

**Published:** 2026-03-10

**Authors:** Wonhyeop Shin, Jihwan Kim, Dohee Kim, Younha Han, James H. Thorne, Youngkeun Song

**Affiliations:** ^1^ Environmental Planning Institute Seoul National University Seoul Republic of Korea; ^2^ Jeju Institute Jeju Republic of Korea; ^3^ Interdisciplinary Program in Landscape Architecture Seoul National University Seoul Republic of Korea; ^4^ Department of Environmental Science & Policy University of California, Davis Davis California USA; ^5^ Department of Environmental Design, Graduate School of Environmental Studies Seoul National University Seoul Republic of Korea

**Keywords:** camera traps, connectivity, fragmented forest, human–wildlife conflict, species distribution model, unmanned aerial vehicles

## Abstract

Human–wildlife conflicts frequently occur at forest–agriculture interfaces, particularly in fragmented landscapes where wildlife movement corridors intersect with farmland. This study evaluated the effectiveness of a short fence in reducing seasonal incursions by wild boar (*Sus scrofa*) and Korean water deer (*Hydropotes inermis argyropus*) into farmland at Baekbong Mountain, Namyangju City, Gyeonggi Province, South Korea, from January 2021 to February 2022. Using camera trap detections and UAV‐derived environmental data, we developed seasonal habitat suitability models with Maxent and conducted connectivity analyses using Omniscape to identify potential movement corridors. A 200 m fence was installed at a predicted hotspot, and additional camera traps were used to monitor changes in wildlife movement. Trails and roads were identified as key environmental variables influencing habitat suitability for both species. After fence installation, the preferred corridor used by wild boar near a mud pool was effectively blocked, whereas Korean water deer continued to access the same location. Seasonal distribution changes were more pronounced for wild boar, with their range expanding beyond the fenced area. Our results suggest that even relatively short fences can effectively deter wild boar movement while having minimal impact on water deer, highlighting the importance of species‐specific ecological considerations when implementing mitigation measures. These findings provide practical insights for farmers and land managers seeking to reduce wildlife incursions and mitigate human–wildlife conflicts in urban‐fringe ecosystems.

## Graphical abstract

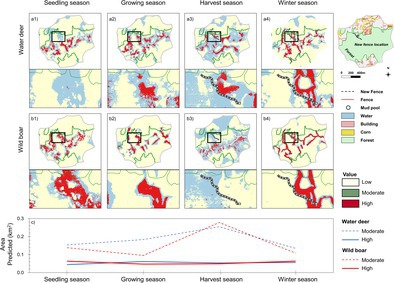

## 1 Introduction

Habitat loss and urban expansion have led to increased human–wildlife interaction in semiurban and urban landscapes (Shochat et al. 2006; Wolf and Ripple 2017).

These interactions are often driven by the increasing fragmentation of natural land cover interspersed with agricultural fields and residual areas created by human activities (Cardille and Lambois 2010; Mitchell et al. 2015). The expansion of urban and agricultural areas also alters landscape structure and functional connectivity between remaining habitat patches (Bradley and Altizer 2007; Partecke et al. 2006). In response to such changes, some animals adapt their behavior, including shifting daily activity timing or metabolic rates, or migrating toward areas with greater food security and refuge (Morelle and Lejeune 2015; Singh et al. 2012). Consequently, several wide‐ranging species have adapted well to urban environments despite reduced connectivity (Crooks 2002; Riley et al. 2003).

To understand wildlife distributions in temperate regions, it is also necessary to consider seasonal variation, since resource availability, interspecific competition, and home‐range dynamics fluctuate with seasonal shifts (Morelle and Lejeune 2015). Therefore, seasonal presence and movement patterns of wildlife should be examined when studying urban forest fringe ecosystems. Various types of fences, including electric fences and deer exclusion grids, have been installed to reduce wildlife incursions and conflicts in urban areas, providing roadkill prevention, biosecurity, and crop protection (Clevenger et al. 2001; Honda et al. 2020; Laguna et al. 2022). Furthermore, research has explored the impact of conservation efforts facilitated by the installation of fences (Pfeifer et al. 2014). To optimize efficiency and minimize costs, hotspot‐based fence placement has been emphasized (Mysterud and Rolandsen 2019). However, the behavioral responses of wildlife to newly erected fences—especially their seasonal movement adjustments—remain poorly understood (Laguna et al. 2022; McInturff et al. 2020). In particular, few studies have addressed how barriers limiting access to seasonal food resources affect wildlife responses (Tolon et al. 2009). To address these gaps, this study aims to examine seasonal changes in habitat suitability and connectivity of water deer ( *Hydropotes inermis* ) and wild boar ( *Sus scrofa* ) around hotspot fencing in a fragmented urban forest fringe. Using camera traps, UAV imagery, and connectivity modeling, we evaluate how fencing influences movement pathways and potential mitigation strategies for human–wildlife coexistence. Electrical circuit theory is a novel tool that has quantified organism movements across multiple potential paths within a landscape, offering substantial advancements in understanding landscape connectivity and animal behavior (McRae et al. 2016; Dickson et al. 2019; Kim and Song 2023). Unlike traditional least‐cost modeling, which identifies only a single optimal route, circuit theory treats the landscape as an electrical network where movement probability flows through all possible pathways, reflecting the cumulative effects of multiple routes (Kim and Song 2023).

Terrestrial mammals are particularly sensitive to barriers since their movements can be directly blocked by fences (Laguna et al. 2022). Wild boar ( *Sus scrofa* Linnaeus) and Korean water deer ( *Hydropotes inermis argyropus* ) are common ungulates in South Korea and are designated as “harmful wild animals” by the Ministry of Environment. Wild boar distribution has expanded in the Northern Hemisphere (Veeroja and Männil 2014). It is found in various habitats but prefers vegetated areas, especially in deciduous forests (Won and Smith 1999). Studies conducted in Korea have indicated that wild boar actively utilize wind‐farm–associated forest areas and nearby management roads, reflecting their behavioral flexibility in response to anthropogenic landscape modification (Kim et al. 2025). Conflicts between wild boar and humans have increased worldwide, causing many problems such as the degradation of native vegetation and spread of infectious diseases (e.g., African swine fever) that can cause significant damage to pig farms (Cwynar et al. 2019; Boklund et al. 2020) and damage to field crops, with negative economic consequences (Barrios‐Garcia and Ballari 2012).

Water deer have Chinese and Korean subspecies (Geist 1998) and prefer dense forests and grasslands and are often found in lowland, mountainous, and waterfront areas (Kim et al. 2011; Won and Smith 1999). Water deer were once abundant throughout Korea until the 1990s when hunting‐based population controls were implemented (Kim et al. 2011). However, the water deer population continues to increase due to low levels of predation (Jo et al. 2018). Empirical evidence also suggests that water deer exhibit distinct behavioral responses to fences and other linear structures, which may influence their spatial distribution near forest–agriculture boundaries (Park et al. 2021). Consequently, water deer are often observed grazing on vegetation in rural villages, in agricultural areas, and even along metropolitan roadsides. Collectively, these patterns indicate that anthropogenic features—such as wind‐farm management roads and fencing—can modify habitat accessibility and movement dynamics of large herbivores in fragmented forest landscapes of South Korea.

This study aims to produce seasonal habitat suitability models for these two species in an area of fragmented habitat through the use of high‐resolution spatial and temporal data from camera traps and unmanned aerial vehicles (UAVs). It will consider crop seasonality and examine changes in the potential corridor used by both species after fence installation in a possible hotspot area. To quantify the functional connectivity of the two species, electric‐circuit‐connectivity modeling analysis based on species distribution modeling was conducted. The results of this study offer critical insights for farmers and wildlife managers in target management areas, elucidating the multifaceted impacts that fence erection can have on different species. This knowledge is particularly important for addressing coexistence challenges and to support comprehensive wildlife conservation strategies related to human–wildlife conflict.

## 2 Materials and Methods

### 2.1 Study Area

**FIGURE 1:**
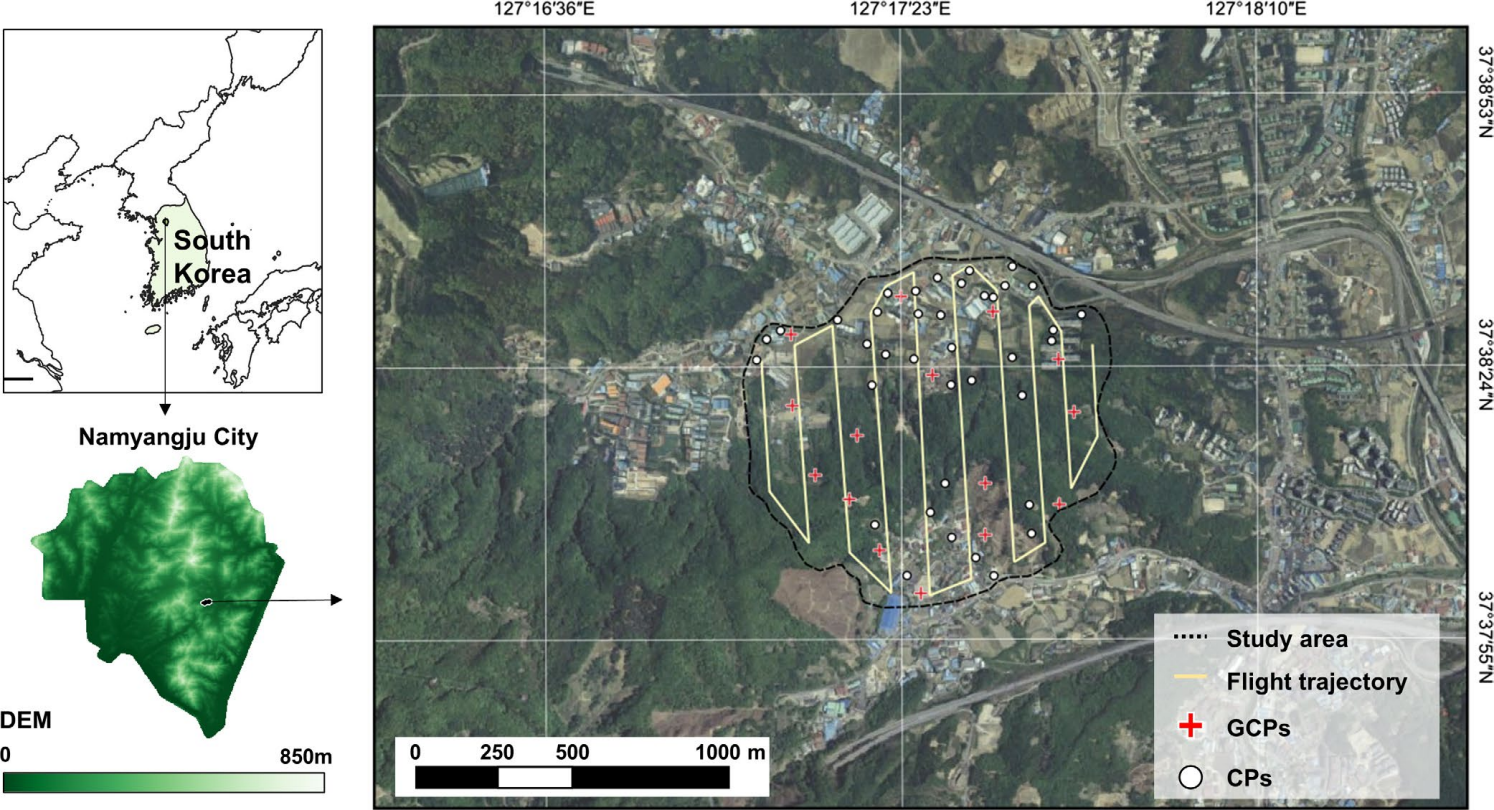
The study area is a fragmented landscape on the edge of Namyangju City, South Korea. The image on the right was taken by the VWorld satellite and shows the trajectory of the UAV flights and the positions of the ground control points (GCPs) and check points (CPs).

The study site was located in a forest‐fringe area of Baekbong Mountain in Namyangju City (which has a total area of 458.1 km^2^; 37°30′–37°46′ N, 127°05′–127°22′ E), Gyeonggi Province, South Korea (Figure 1). The site was selected because it is a fragmented forest with accessibility to agricultural fields, which facilitated the study of fence installation and its ecological impacts. Forest fragmentation was confirmed using VWorld satellite imagery (Figure 1). A short section of fencing already existed along the forest–field boundary, and an additional 200 m experimental fence was installed to assess species‐specific responses. The surrounding landscape includes a mud pool, small water bodies, and informal trails that may influence wildlife movement (Figure 3). The climate is hot and humid in summer and cold and arid in winter under the East Asian monsoon system. The annual mean temperature is 21°C, with monthly means ranging from −4.2°C in January to 25.0°C in July. Vegetation consists primarily of mixed deciduous broadleaf and needleleaf forest naturally dominated by pine ( *Pinus densiflora* ) and nut pine ( *Pinus koraiensis* ), which serve as food resources for local wildlife. Nearby grassland and cropland form the main nonurban land‐cover types, and lettuce, green onion, pepper, corn, potato, sweet potato, and beans were identified as the main crops through direct field surveys and ground verification aided by UAV imagery interpretation. These surveys were conducted once per season within a 1 km buffer around all camera trap sites to capture temporal changes in agricultural patterns.

### 2.2 Occurrence Data Collection and Spatial Filtering

Camera traps were deployed at 18 fixed locations for 14 months (January 2021–February 2022) and remained in place throughout the survey period. The cameras were positioned within regular hexagonal cells with 100‐m‐long sides to ensure even spatial coverage (Figure 1). Camera trap locations were selected based on traces of wild boar, such as rubbing trees, mud pools, soil rooting, and feces, or along wild animal pathways, to capture multiple events during the period. Cameras were located at least 50 m from each other. Cameras with infrared flashes, types D3 and D9, were used (Hong Kong Ica Industry Co., China). These were tied high on tree trunks beyond the reach of wild boar (1.18 m, SD = 0.20 m) to ensure safety (Appendix S1: Table S1). Both camera types had an aperture angle of 90° and detected animal movement using a passive infrared sensor (PIR sensor) with a detection range of ≤ 20 m. Camera PIR sensitivity was set to the normal level from among three choices (low, normal, and high). When triggered, they recorded 30 s of continuous video (resolution: 1920 × 1080 pixels). The cameras were checked and their memory cards replaced every 2 weeks.

**FIGURE 2:**
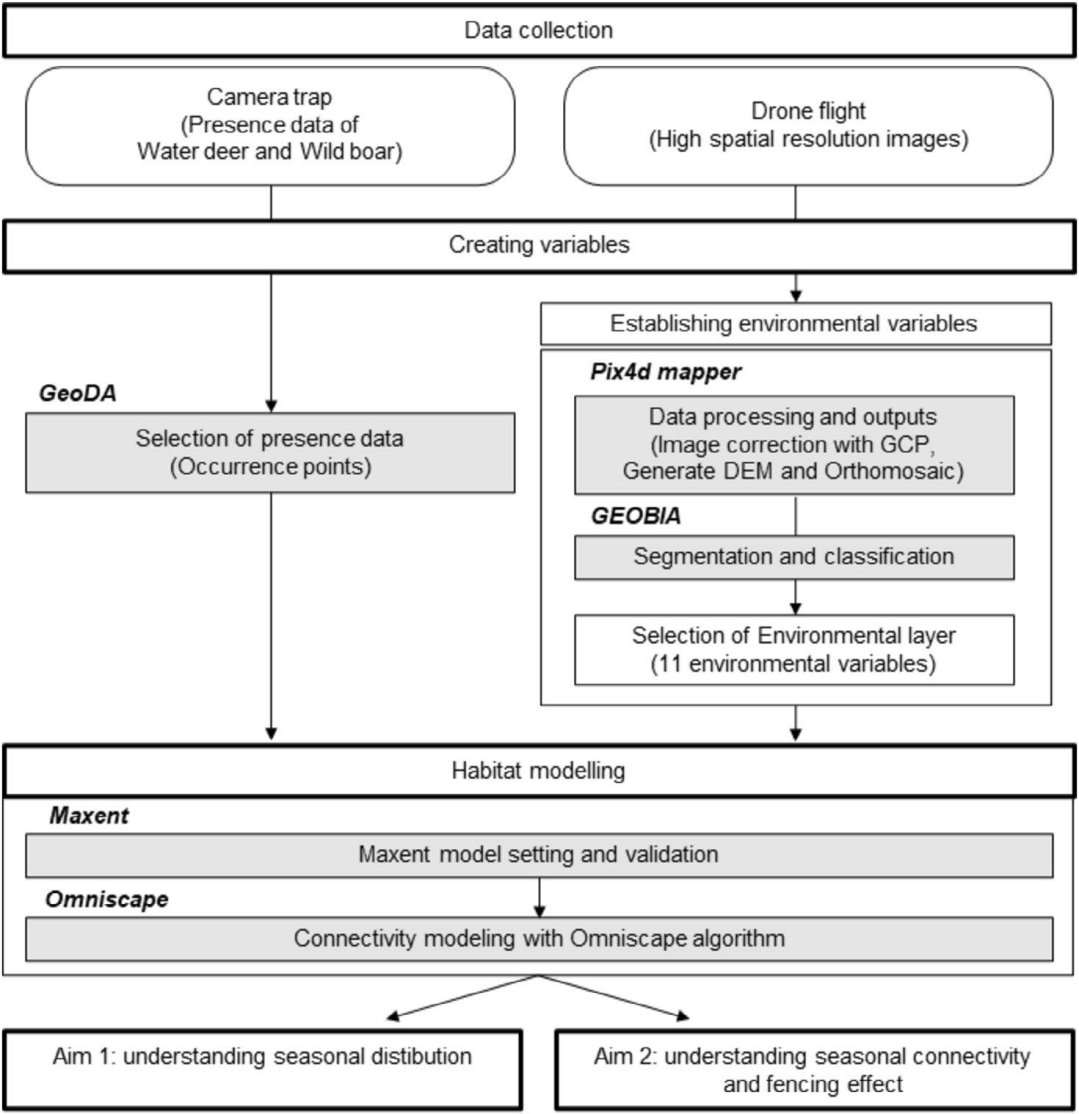
Flowchart of data and processes used in this study to estimate seasonal predicted distribution, connectivity, and the effect of fencing.

The cumulative monitoring effort amounted to 7560 trap‐nights (18 cameras × 420 days). During the representative months of each crop‐growth stage, the camera traps recorded 283, 382, 694, and 1209 water‐deer detections in the seedling (March), growing (June), harvest (September), and winter (December) seasons, respectively. We selected these four months as representative phenological periods corresponding to distinct stages of crop and vegetation cycles in the study area. Such an approach has been commonly used in studies of ungulate seasonal movement and crop–wildlife interactions (Morelle and Lejeune 2015). Local Moran's I applied based on a distance weight of 160 m and a 164 power value of 2 in GeoDa (version 1.20) and spatial autocorrelation of the records was removed (Kwon et al. 2016). This statistic was chosen because it identifies both local clusters and outliers, reducing pseudo‐replication among nearby camera traps while retaining ecologically meaningful hotspots, and provides the fine‐scale sensitivity needed in fragmented landscapes with heterogeneous sampling density (Dormann et al. 2013). After spatial filtering, 981 and 146 detections by water deer and wild boar, respectively, were retained in the final dataset used for habitat suitability modeling (Figure 2). Occurrence points for utilization in species distribution models were randomly generated within a circular sector area that considered camera trap shooting range, 90° camera angle, and a detectable range of up to 20 m. To decrease the spatial aggregation, one occurrence point was randomly selected per pixel per 1 m.

### 2.3 Environmental Predictors of Habitat Modeling

To establish environmental variables, high‐resolution UAV images were acquired in June 2021 during morning hours (09:00–11:00 a.m.) under clear‐sky conditions using a DJI Phantom 4 Pro V2.0 (DJI, Shenzhen, China) equipped with a built‐in ground control point (GCP) unit and a 1‐in. 20 MP CMOS sensor (5480 × 3648 pixels) with an 84° field of view and storage of 4K images at 60 fps. The flight mission was planned using DJI Go 4 and the Pix 4D capture applications. Altitude was set at 150 m and flying speed to 5 m/s, yielding a ground sampling distance of 7.85 cm/pixel. To generate the orthomosaic, a front and side overlap of 80% was set between the images to ensure coverage of every part of the study area. This resulted in 162 collected images. Image correction was conducted using GCP coordinate values, which were manually measured using the Gaia GPS app. Non‐natural constructs, such as grave headstones, centers of manhole covers, and corners of road paint located in low‐slope open spaces, were selected as reference locations because they are permanent, geometrically stable, and easily identifiable features that do not change seasonally. These points provided greater positional consistency and accuracy than natural landmarks that could be affected by vegetation growth or weather conditions. A total of 15 GCPs and 39 check points (CPs) were used to assess the absolute accuracy of orthomosaic model. The digital elevation model (DEM) and orthomosaic were produced based on the structure from motion algorithms using the UAV imagery and Pix4Dmapper software (www.pix4d.com). The resulting DEM and orthomosaic were aligned to each other and used to generate environmental variables.

All environmental layers derived from UAV imagery were resampled to 1 × 1 m resolution to align with the ground‐sampling distance and to preserve fine‐scale habitat features such as informal trails and crop boundaries. A preliminary sensitivity test using 0.5 and 2 m resolutions indicated that 1 m provided the best trade‐off between spatial accuracy and computational efficiency, avoiding loss of micro‐habitat details while ensuring stable model performance, and 13 distance‐related variables in four types of habitats were selected. Environmental predictors were selected to represent key ecological factors influencing ungulate habitat use, including forest composition (oak, conifer, forest edge: Kim et al. 2011), food resources (sweet potato, corn: Morelle and Lejeune 2015), anthropogenic features (roads, trails, fences: Laguna et al. 2022), water availability (mud pools, water bodies: Ito et al. 2024), and topography (elevation, slope, roughness: Singh et al. 2012). We focused on sweet potato and corn, the dominant field crops and primary foraging resources for both wild boar and water deer in the study. These predictors were represented as Euclidean distances to the corresponding landscape features. To reduce the effect of potential multicollinearity on the models, Pearson's correlation coefficient was calculated and only the variables < 0.75 were selected (Dormann et al. 2013). Therefore, from the 16 environmental variables, 11 were selected to predict the distributions of the species. These consisted of oak tree, coniferous tree, and edge of forested area (forest); corn (crop); distances to roads, informal trails, and fences (human‐related); DEM, slope, and roughness (topography); and distance to water bodies (hydrography) (for further details, see Appendix S1: Tables S2 and S3).

### 2.4 Maxent Model Setting and Model Validation

To model seasonal predicted distributions for water deer and wild boar, maximum entropy species distribution modeling (Maxent, version 3.4.4) was used. This approach applies the principle of maximum entropy to relate presence‐only data to environmental variables, which is particularly suitable for camera‐trap datasets with limited or spatially biased detections (Phillips et al. 2009; Elith et al. 2011). We used Maxent because it shows robust performance with small sample sizes, allows incorporation of continuous predictors, and produces ecologically interpretable response curves, making it one of the most widely used algorithms for mammalian habitat modeling (Merow et al. 2013). In this study, presence data of water deer and wild boar were collected by camera traps as a representative value for each hexagon, and environmental layers were derived from high‐resolution mapping resulting from the use of UAVs (Figure 2).

The presence data was solely captured through camera traps; therefore, a bias grid file was created by assigning a sampling probability value of 1 within the camera trap shooting range (90° angle and 20 m detection distance) and the value of 0.01 to other areas to restrict the background sampling area (Vollering et al. 2019). The output format was set to cloglog as it provides a better result compared with logistic when bias correction is used (Phillips et al. 2017). The final output provides relative suitability values from 0 (unsuitable) to 1 (suitable). All other parameters remained on default settings. The K‐fold cross‐validation method was used to fit the model (Fielding and Bell 1997), with occurrences partitioned into four folds (k = 4), allocating 75% of the data for training and 25% for testing in each run. Background points were sampled within the drone‐surveyed study area shown in Figure 1, while a value of 0.5 indicated that the model prediction was only as good as a random guess (Fielding and Bell 1997). The final probability model was transformed into a binary map using the maximum of the sum of training sensitivity and specificity, with the value above for presence and under for absence (Manel et al. 2001). Thus, eight models representing the two species and four seasons (seedling, growing, harvest, winter) were obtained.

### 2.5 Connectivity Modeling

Functional connectivity analysis was applied to evaluate the effects of fencing on wildlife because it represents movement potential and dispersal capacity within human‐modified landscapes, capturing how animals navigate permeable barriers (Cushman et al. 2009). We implemented this analysis using open‐source Julia software (version 1.6.5) and the Omniscape algorithm, a circuit theory–based, omnidirectional connectivity model that estimates potential pathways across heterogeneous landscapes. Omniscape calculates current flow between focal and neighboring pixels within a circular moving window, treating the landscape as a resistance surface that reflects multiple, simultaneous movement routes rather than a single least‐cost path, thereby providing a more realistic depiction of connectivity in fragmented habitats (McRae et al. 2008; Dickson et al. 2019). Species distribution maps, generated using Maxent, were imported into Omniscape to generate the input spatial source and resistance layer (see Section 2.4). The habitat suitability model was employed as the source layer and was transformed to produce the resistance layer by reversing the values in order to allocate high movement resistance to low‐quality areas (1‐suitability) (Costa et al. 2021).

Two Omniscape outputs were generated to represent complementary aspects of landscape connectivity. The first, cumulative connectivity, represents the total potential movement intensity accumulated across the landscape, where higher values indicate areas that contribute more strongly to overall movement. The second, normalized flow potential, was obtained by dividing cumulative connectivity by current flow under a no‐resistance condition, thereby indicating how movement is concentrated (values > 1) or diffused (values < 1) relative to an unrestricted flow baseline. The normalized flow potential map was subsequently reclassified into three categories: low (impeded < 1), moderate (diffused = 1), and high (channeled or intensified > 1). Resistance surfaces were generated through a linear transformation of suitability values, following the approach commonly used in circuit‐theory connectivity modeling. Omniscape was then run using a moving‐window radius of 100 pixels and a block size of 3, which determine the local neighborhood through which omnidirectional current flows are computed. These outputs effectively highlighted the primary movement zones for water deer and wild boar and helped visualize how fencing altered their connectivity patterns. To compare seasonal connectivity patterns within and between species, the spatial overlap in the cumulative connectivity map was assessed using pairwise Schoener's D metric with ENMTools in R, version 4.1.3 (Warren et al. 2010). *D* statistic values range from 0 to 1, where 1 corresponds to identical niche overlap or close to the absence of overlap, and 0 corresponds to no niche overlap.

### 2.6 Fence Erection in Possible Hotspot Area

**FIGURE 3:**
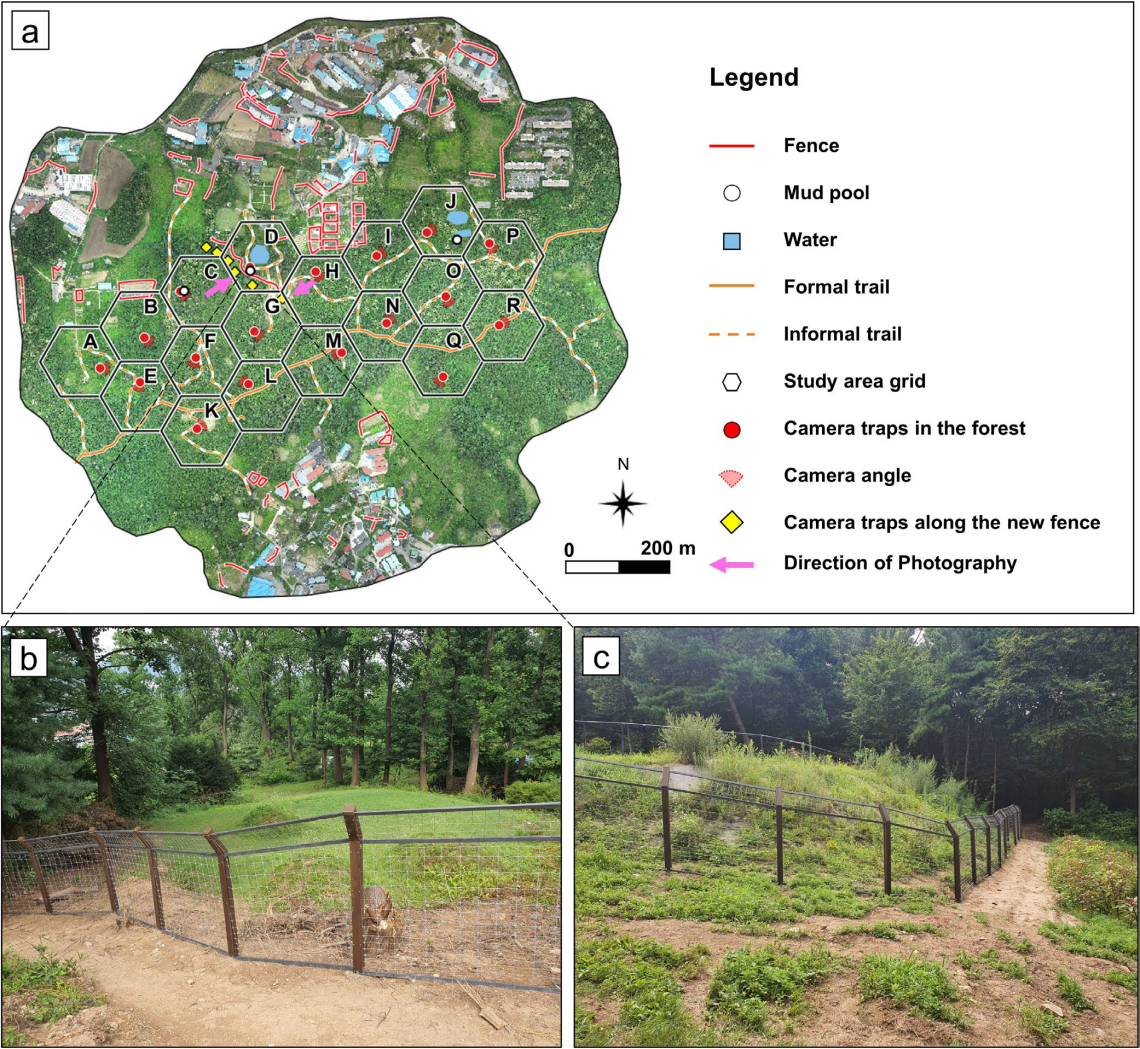
Aerial view captured by UAVs in cell range of camera traps in the study site (a) and photographs of fences (b and c).

To observe the effect of fencing in water deer and wild boar possible hotspot areas, fencing was erected considering the following factors: (1) a location where the probability of presence and connectivity is relatively high before fencing erection; (2) the presence of a mud pool, which is a preferred habitat; and (3) consultation with the land owner. In addition, there were three aspects of fence construction that had to be considered in its design since large mammals pass through fences by three methods: jumping over the fence, lifting up and crawling under the fence, and penetrating weak areas of the fence material. Therefore, the fence was designed and installed considering these three aspects (Figure 3). Firstly, the upper part of the fence included an approximately 60° angle toward the direction from which animals mostly come. Secondly, the lower part of the fence net was tied with poles to prevent animals from bending or twisting the net and widening gaps. Lastly, the net was comprised of steel wire strong enough not to be broken by animals pushing against it. The fence was constructed of steel wire strong enough to resist pressure from large mammals and had an approximate height of 2 m with a mesh size of about 15 cm. The erected fence was 200 m in length and was installed from July 26 to August 6, 2021. From June 28 to December 31, 2021, six cameras of type D3 facing the possible hotspot area were evenly deployed along the fence to observe approaching wild boar and water deer. During the winter, when crops are not generally damaged by wildlife, a door installed in the middle of the fence was opened throughout the winter season (December–February), during which water deer were occasionally recorded by camera G, located near the door opening as shown in Figure 3.

## 3 Results

### 3.1 Responses to Environmental Gradients

In general, the habitat quality models performed well, with AUC values ranging from 0.933 to 0.950 for wild boar and from 0.857 to 0.964 for water deer. Although AUC varied slightly among seasons, all values exceeded 0.85, indicating consistently strong model performance across seasonal models (Table 1). The two most important variables across all seasons for both species were informal trails and roads, which are both human‐made variables (Table 2). However, informal trails showed a higher probability of occurrence as movement pathways, whereas for roads, the distribution appeared to be more dispersed, maintaining a certain distance. During all seasons except the harvest season, water deer primarily used trails. During the seedling season, highly suitable areas for water deer decreased, with distances from informal trails extending from 0 to 250 m. Suitable areas were also found at a relatively high elevation (190 m; Appendix S1: Figure S1). During the growing and winter seasons, highly suitable areas for water deer also decreased with distances from informal trails extending from 0 to 50 m and increased with distances from roads of 100 to 300 m. During the harvest season, the greatest contributing variable to the distribution of water deer was the distance to the road (41.66%), with high suitability from 50 to 200 m. Accordingly, during the harvest season, the probability of the presence of water deer increased at locations 50 m to 100 m closer to the village roads than during the growing season.

**TABLE 1:** AUC ± SD values of the different models in Maxent for Water deer and Wild boar.

	Seedling season	Growing season	Harvest season	Winter season
Water deer	AUC = 0.950 ± 0.012 (*n =* 129)	AUC = 0.941 ± 0.007 (*n =* 182)	AUC = 0.933 ± 0.007 (*n =* 350)	AUC = 0.941 ± 0.018 (*n =* 44)
Wild boar	AUC = 0.932 ± 0.013 (*n* = 101)	AUC = 0.964 ± 0.010 (*n =* 56)	AUC = 0.857 ± 0.063 (*n =* 25)	AUC = 0.926 ± 0.021 (*n =* 28)

*Note:* The details between brackets are the number of occurrences used to train the model.

**TABLE 2:** The Maxent permutation importance analysis revealed the percentage of variable contributions to model construction, utilizing data extracted by the UAVs.

Variable		Water deer				Wild boar			
		Seedling season	Growing season	Harvest season	Winter season	Seedling season	Growing season	Harvest season	Winter season
Forest	Oak	2.32	**9.57**	0.08	2.62	6.04	0.39	3.04	0.58
	Conifer	0.29	0.84	2.75	2.07	0.23	2.40	2.81	1.68
	Edge	1.05	8.87	0.86	**11.42**	4.44	5.54	0.26	1.83
Crop	Corn	**7.80**	5.40	**14.23**	0.26	**17.38**	7.20	**12.68**	0.89
Human	Road	1.15	**25.25**	**41.66**	**39.25**	**11.56**	**10.79**	0.62	**18.87**
	Trails	**36.35**	**29.88**	4.68	**31.61**	**51.50**	**41.45**	3.34	**69.98**
	Fence	6.32	6.73	5.78	3.82	4.64	0.12	**51.52**	1.72
Topography	DEM	**42.02**	2.78	**14.33**	0.51	1.89	0.20	**16.41**	0.00
	Slope	0.18	5.63	3.42	3.13	0.99	0.37	0.03	0.99
	Roughness	0.64	0.36	0.05	0.05	0.53	0.11	1.60	0.22
Hydrography	Water	1.89	4.68	12.15	5.25	0.81	**31.43**	7.69	**3.23**

*Note:* The three variables with the highest contributions during each season and for each species are presented in bold.

During the seedling, harvest, and winter seasons, the distance to trails was the primary distribution constraint for wild boar, with habitat suitability sharply decreasing beyond 50 m from the trails (Table 2; Appendix S1: Figure S2). In addition, during these three seasons, distance to the road was also a primary distribution constraint on wild boar, with high suitability that increased beyond 50, 150, and 140 m from the road, respectively. It was observed that following the growing season, wild boar preferred core areas more than the forest edge. During the harvest season, the distribution of wild boar was strongly influenced by the fence factor, showing an optimum suitability between 100 and 300 m.

### 3.2 Seasonal Predicted Distribution Change

**FIGURE 4:**
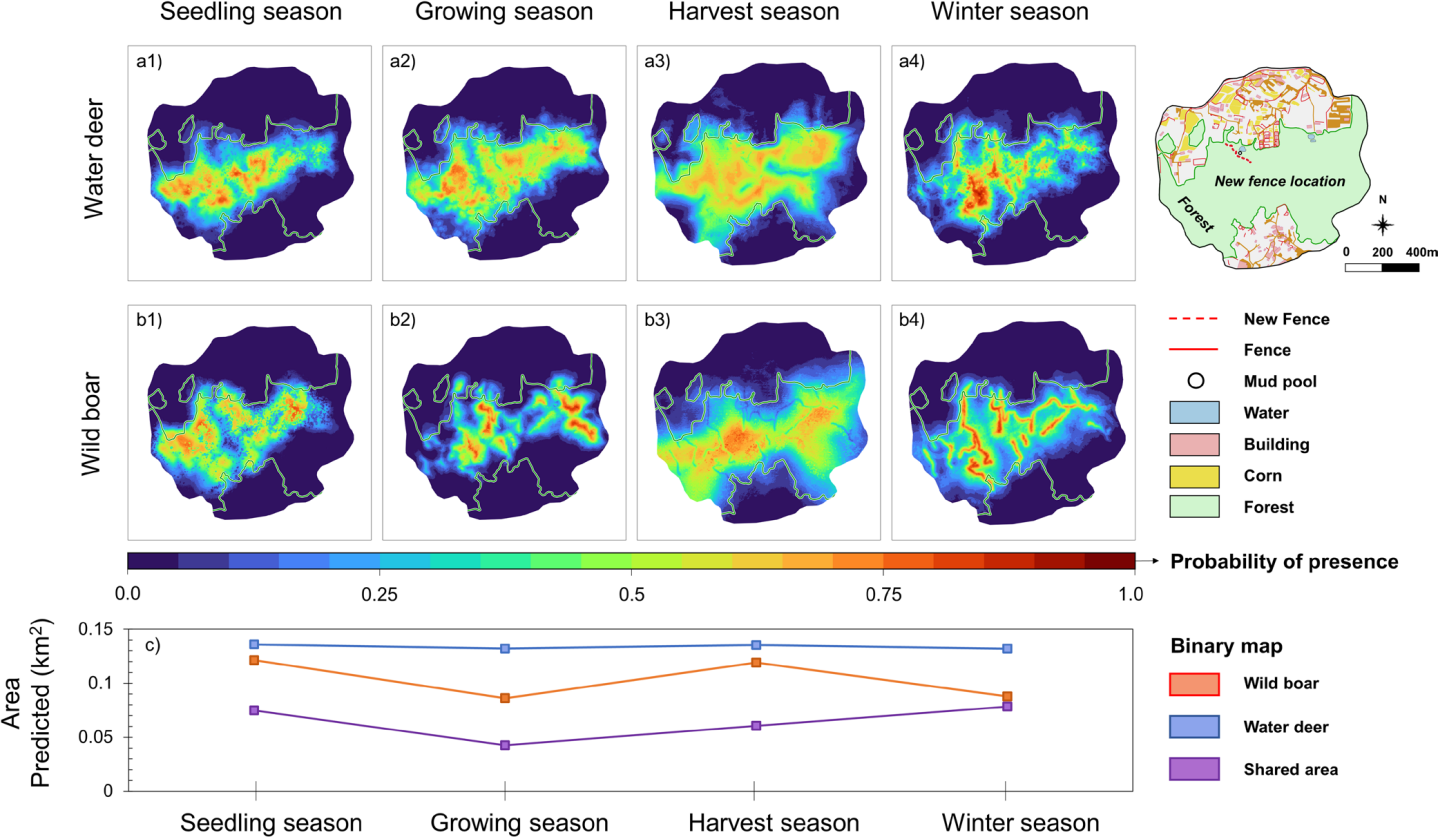
Species distribution model trained on water deer (a) and wild boar (b) and predicted area of binary map (c) for crop rotation showing seedling season (March), growing season (June), harvest season (September), and winter season (December) in fragmented areas.

The distribution of water deer presence‐suitability values throughout the overall study area gradually increased from the seedling season to the harvest season (Figure 4a). In contrast, wild boar showed a different yearly distribution pattern, with high suitability values concentrated around informal trails and water bodies during the growing season and widely distributed during the harvest season (Figure 4b). Across the four seasons, the suitable area (binary presence area) of water deer changed only slightly (within ±4.3%), whereas the suitable area for wild boar fluctuated markedly—decreasing by 29.1% during the growing season, increasing by 38.5% during the harvest season, and decreasing by 26.4% during the winter season (Figure 4c). This suggested that the seasonal predicted distribution of wild boar was more dynamic than that of water deer. The shared suitable areas for both species decreased by 43.3% from the seedling season to the growing season and then gradually increased (Figure 4c, Appendix S1: Figure S3). Both species were concentrated near informal trails during the winter season (Figure 4c).

### 3.3 Seasonal Connectivity Changes and Fencing Impacts

**FIGURE 5:**
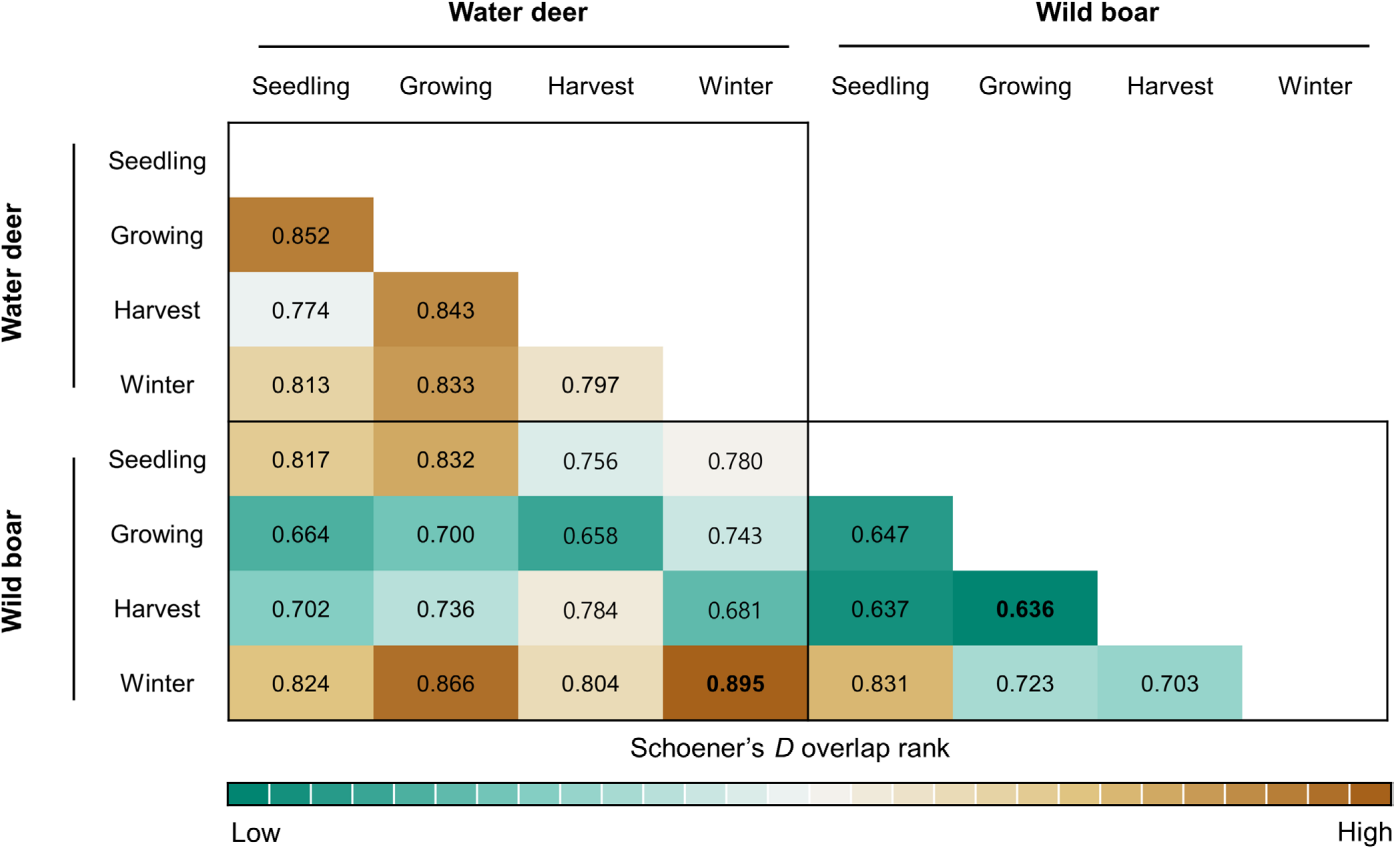
Schoener's *D* overlap index (higher values mean similar functional connectivity) between pairs of species in the connectivity model values made using Omniscape. Highest and lowest ranks in bold, ranging from 28th (green) to 1st (brown).

**FIGURE 6:**
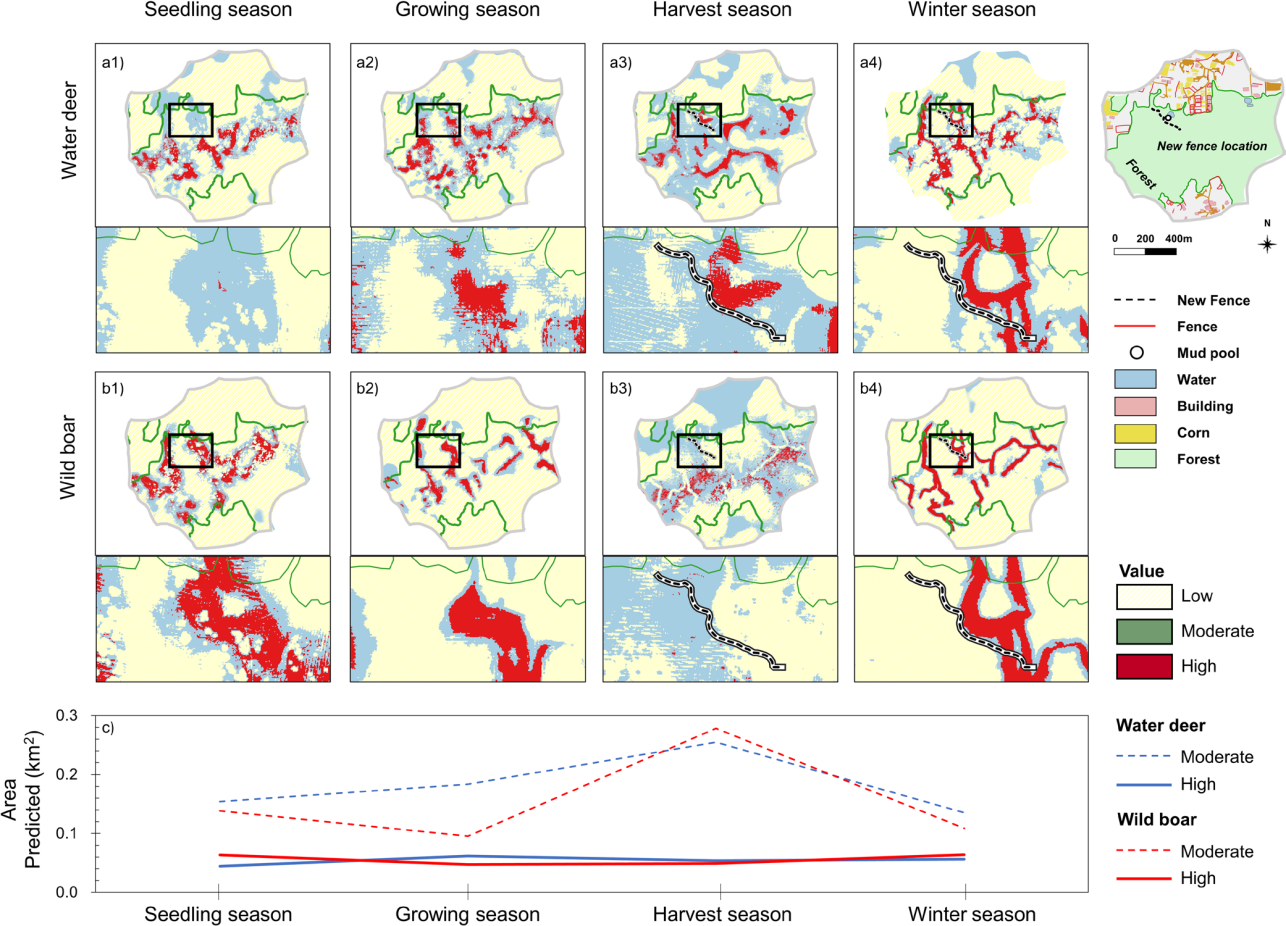
Normalized flow potential connectivity analysis for water deer (a), wild boar (b) except buffer zone (100 m), and changed predicted area for both the species according to the reclassified level of connectivity (c) for crop rotation within the mountain area.

The cumulative landscape connectivity similarity across seasons for wild boar showed generally low ranks (green color) and water deer showed high ranks, according to Schoener's *D* metric (brown color) (Figure 5). The metrics showed that wild boar predicted movement patterns sharply changed depending on the season, particularly during the growing and harvest seasons, and became similar during the seedling and winter seasons. Water deer predicted movement patterns did not change notably seasonally. The metric of overlap also exhibited the highest overlap rank among the two species during the winter season (Figure 5). This meant that both species had similar predicted movement patterns during winter, with trails forming similar potential corridors that facilitated their movement (Figure 6a4,b4).

In the connectivity maps of the two species, the fencing effect was found to block the potential corridor (high normalized flow potential connectivity value) of wild boar (Figure 6b2,b3). The modeled connectivity showed a change of spatial pattern near the fencing, which was substantially different for both species during the harvest season. From the growing season to the harvest season, the moderate‐permeability area increased by 38.8% and 191.8% (area‐based) for water deer and wild boar, respectively (Figure 6c). However, at the fence erection location around the mud pool, the preferred habitat of wild boar, the high permeability area of wild boar declined, while it remained the same for water deer during the harvest season (Figure 6a3,b3). After fencing was erected, wild boar was not detected on camera trap D at the mud pool, but water deer continued to be recorded. During the winter season, there was no connectivity difference observed between the two species, especially at the fence because the fence gate might be open during the winter (Figure 6).

## 4 Discussion

### 4.1 Habitat Model Using High‐Resolution UAV Data

These results show that the detection of the ecological and artificial attributes of wildlife habitat conditions from UAV images enables species distribution modeling within a small‐area landscape. The high‐resolution environmental variable data acquired from UAVs provided important contributions to the models, such as distances to trails and fences, DEM, and distances to crops (Table 2), and the provision of high‐resolution images from UAV‐based remote sensing platforms has been recently suggested for the effective and efficient monitoring of medium‐to‐large mammals and their habitats (Mangewa et al. 2019). Although mid‐ or high‐resolution imagery is freely obtainable from aircraft or satellites (Kim and Daigle 2012), the resolution and accuracy of those measurements are lower compared with UAV field imagery. In addition, sub‐meter resolution data from satellite or aerial platforms can be expensive. This study found that the use of UAV imagery was particularly suitable for detecting small‐ and medium‐sized environmental and disturbance features in landscapes, which is important for understanding wildlife habitats (Ezat et al. 2018). Structures and disturbances related to human‐induced environmental features can be detected through classification approaches or through visual inspection of orthomosaic imagery.

Nevertheless, few previous studies have focused on species distribution models using high‐quality data derived from orthomosaic drone imagery, such as trail proliferation. In this study, trails were found to most constrain the distributions of water deer and wild boar, while distance to fences was an important environmental variable affecting the presence of wild boar during the harvest season (Table 2). Establishing detailed human‐induced parameters can be helpful in understanding the distribution of wild animals in urban forest fringe areas. Furthermore, elevation data from a DEM and classified maps from UAVs can elucidate the effects of human impacts on landscapes (Ancin‐Murguzur et al. 2020). For example, in this study, UAV data were used for DEM generation and vegetation classification was performed on the UAV orthomosaic using object‐based image analysis to extract coniferous and oak tree canopy data, with an overall accuracy of 86.17% (Appendix S1: Table S3). Generally, the DEM had a higher contribution to the models for water deer and wild boar compared with the impact distance to oak tree variable (Table 2). This may suggest that the influence of informal trails used by wildlife is more substantial than the function of oak as a food source in urban forest edge areas. This pattern likely reflects the context of urban‐fringe environments, where human disturbance and accessibility shape wildlife movement more than food availability. While similar dynamics may exist in other fragmented or peri‐urban landscapes, further comparative research is needed to determine the generality of this finding. Depending on the target species, additional spatial data such as roads and artificial waterways constructed through the use of UVA generated data can also be helpful in building species distribution models (Yokochi et al. 2015). Furthermore, seasonal animal predicted movement patterns where high‐resolution data or specific structures are required can be effectively analyzed by combining UAV and time‐series camera trap data. However, despite the advantages of UAV‐based orthomosaics, there are several conditions to consider. First, trade‐offs may be necessary between the desired resolution and the time required for mapping an area (Cruzan et al. 2016). Second, the features to be detected have to be directly visible from the air in order to be identified in an orthomosaic. Furthermore, many methodologies for image–object segmentation and classification should be considered in order to increase the accuracy and reliability of the species distribution modeling (De Luca et al. 2019; Kucharczyk et al. 2020). In addition, recent studies highlight that integrating LiDAR and hyperspectral sensors on UAV platforms can overcome some limitations of optical UAV imagery by providing detailed three‐dimensional vegetation structure and fine‐scale spectral information for species or habitat discrimination (Aasen et al. 2018; Gu et al. 2022). However, these systems require higher payload capacity and precise sensor registration, which can limit their use for large‐area monitoring and increase operational costs.

### 4.2 Target and Possible Hotspot‐Oriented Fencing Strategy

Erecting fencing in fragmented areas near human development can serve nature conservation or protect crops and humans from wildlife incursions (Clevenger et al. 2001; Laguna et al. 2022; Xu et al. 2021). However, to date, the actual movements of species following the installation of fencing relative to these applications remain rarely documented (McInturff et al. 2020). A fence installed without consideration for the movement of species in such areas could lead to several problems. First, ungulates can easily move from a damaged area to other nearby fields that can then result in either the concentration or spread of crop damage (Geisser and Reyer 2004). Second, a fence installed for only a few target species may cause conservation‐related problems for the movement of nontargeted species, including endangered animals (McInturff et al. 2020). These side effects can be reduced by strategic selection of fence design, length, and location, and recent research has examined the importance of understanding possible hotspot fencing, a practical method to effectively reduce the transit of wildlife using a short fence (Mysterud and Rolandsen 2019). In particular, this approach is highly usable in applications that require prediction and interruption of the movement paths of wild animals (Gooding and Brook 2014). Furthermore, this method can suggest solutions through the use of predictive modeling, especially when potential corridors are uncertain.

The length of wildlife fencing may become a contentious issue, due to its impact on conservation (Bode and Wintle 2010; Linnell et al. 2016). Long fences are installed in regions to prevent the spread of wildlife diseases and reduce conflicts with humans. Fence lengths of at least 5 km have been suggested to improve human safety (Huijser et al. 2016; Mysterud and Rolandsen 2019; Rytwinski et al. 2015). However, long fences do not necessarily guarantee or facilitate higher use by wildlife of underpasses, as use greatly varies between locations (Huijser et al. 2016). In addition, the installation of long fences in urban forests can be difficult due to restrictions on privately owned land. Furthermore, artificial and natural barriers such as steep slopes, rock cliffs, and high walls also have substantial impacts on wildlife movement (Cozzi et al. 2013) that can render a long perimeter fence less effective. In this study, although the fence was only 200 m in length, it successfully reduced the distribution of wild boar in a forest edge area (Figure 4) and decreased connectivity (potential value of normalized flow) from high to low (Figure 6) around the fencing location. The wild boar distribution area sharply increased after fence erection, while that of water deer gradually increased, suggesting that the species had to disperse across more habitats once access was blocked to a high‐habitat value location (Figure 4).

Furthermore, at the new fence erection location around the mud pool, a preferred habitat for wild boar, the high‐permeability area of wild boar disappeared, while high permeability was maintained for water deer during the harvest season (Figure 6). This pattern indicates that the fence more strongly affected wild boar movement, as fences are known to restrict large mammals' access to agricultural foraging areas and alter their spatial use patterns during food‐rich periods (McInturff et al. 2020; Jakes et al. 2018; Laguna et al. 2022). Once access to crop fields was blocked, wild boar likely shifted their foraging range toward adjacent forested areas, whereas water deer, which rely more on forest edges, were less influenced by the barrier.

Therefore, we found that the effectiveness of possible hotspot fencing may vary depending on the species' behavioral traits and their dependence on agricultural food resources.

Following fence erection, the effect of possible hotspot fencing on the movement of wild boar and water deer was monitored, and the differences in their predicted movement patterns were observed using the camera traps installed along the fence and camera D at the mud pool (Figure 3). The fence was found to have blocked the access of wild boar to crops and the mud pool, and they were seen to go back or walk along the fence, but the herd of wild boar that used to visit the mud pool was not detected there following the installation of the fence. In addition, fence‐end effect, a concentration of animal crossings at fence ends (Huijser et al. 2016), was not identified for wild boar through the camera traps at the edge of the hotspot fencing. In contrast, water deer crossing was detected at the camera traps at the mud pool, traces of water deer collisions with the fence were found, and water deer were observed walking along the fence by six camera traps deployed along the fence. Therefore, even if fencing length is short, if it is properly designed based on the requirements of the target species, this approach can reduce their incursions. To mitigate potential effects on non‐target species, wildlife‐friendly fencing designs—such as partial openings, adjustable heights, or small passage sections—can be incorporated. Moreover, combining hotspot fencing with underpasses or natural gaps could preserve essential movement routes for non‐target fauna while maintaining agricultural protection. This strategy can be considered in several aspects. First, fencing can be designed considering the physical features of the target species in order to separate them in an ethical manner. Although this study provides new insights into species‐specific responses to fencing, several limitations should be noted. The monitoring period was limited to one year and a single fence site, which may not fully capture temporal variability or long‐term behavioral adaptation. In addition, camera‐trap detections were constrained by vegetation density and topography, which could have influenced detection probability and spatial accuracy. Future research should therefore expand to multi‐year and multi‐site monitoring across various habitat types to evaluate how fencing effectiveness changes under different ecological and landscape conditions. The integration of advanced monitoring technologies, such as UAV‐based mapping, automated image recognition, and GPS tracking, can improve behavioral understanding and model precision. Moreover, comparative studies that evaluate fencing alongside alternative or complementary interventions—such as habitat restoration, wildlife corridors, or adaptive crop management—would provide a more comprehensive understanding of human–wildlife coexistence strategies (Jakes et al. 2018; McInturff et al. 2020). These efforts will contribute to developing adaptive, species‐specific, and socially acceptable fencing strategies that minimize human–wildlife conflict while maintaining ecological connectivity. Furthermore, incorporating socio‐ecological perspectives and stakeholder engagement can enhance the practical applicability and long‐term sustainability of fence‐based management in fragmented landscapes.

## 5 Conclusions

The findings of this study confirm, through the use of noninvasive survey data, camera traps, and UAVs, the performance differences of fencing on water deer and wild boar at a possible hotspot area in an urban forest fringe area. The high temporal and spatial resolution data obtained from this equipment was used to establish well‐performing seasonal habitat suitability and connectivity models. The distributions of water deer and wild boar were found to be mainly affected by trails and roads. Fencing is an important environmental variable that affects wild boar movement. By means of a comparative approach between seasonal suitable wildlife habitat and a connectivity map of target species, the shift in wild boar movement to a nearby core area and the loss of a primary corridor to its preferred habitat due to fencing at the possible hotspot area have been highlighted. The change in connectivity among water deer was not considerable compared with that of wild boar near fencing. However, long‐term monitoring following the erection of fencing and studies from other sectors and involving other target species could potentially better elucidate its effects on wildlife. Moreover, the integrative workflow combining UAV‐based habitat mapping, species distribution modeling, and connectivity analysis is transferable to other regions and taxa, enabling context‐specific designs that balance conflict reduction and ecological function. These findings provide actionable guidance for mitigating human–wildlife conflict in agricultural landscapes: short, hotspot‐oriented fences at key corridors, coupled with seasonal gate management, can reduce crop damage while maintaining necessary permeability for non‐target movements. Understanding the effects of hotspot fencing in fragmented areas may help farmers and wildlife managers to better allocate local and regional management efforts to solve coexistence issues and establish wildlife conservation strategies.

## Author Contributions

**Wonhyeop Shin:** conceptualization (equal), data curation (lead), formal analysis (lead), funding acquisition (supporting), investigation (lead), methodology (lead), project administration (lead), resources (lead), software (lead), supervision (equal), validation (lead), visualization (lead), writing – original draft (lead), writing – review and editing (equal). **Jihwan Kim:** conceptualization (supporting), data curation (supporting), formal analysis (supporting), methodology (supporting), resources (supporting), writing – original draft (supporting), writing – review and editing (supporting). **Dohee Kim:** conceptualization (supporting), investigation (supporting), methodology (supporting), resources (supporting), visualization (supporting), writing – original draft (supporting), writing – review and editing (supporting). **Younha Han:** data curation (supporting), formal analysis (supporting), investigation (supporting), validation (supporting), visualization (supporting), writing – original draft (supporting), writing – review and editing (supporting). **James H. Thorne:** conceptualization (supporting), methodology (supporting), supervision (supporting), writing – original draft (supporting), writing – review and editing (supporting). **Youngkeun Song:** conceptualization (lead), funding acquisition (lead), methodology (supporting), project administration (lead), resources (lead), supervision (lead), writing – original draft (equal), writing – review and editing (equal).

## Conflicts of Interest

The authors declare no conflicts of interest.

## Supporting information


**Data S1:** ece373000‐sup‐0001‐Supinfo.docx.

## Data Availability

I confirm that the Data Availability Statement is included in the main file of my submission and that all necessary data files are accessible to editors and reviewers (https://doi.org/10.5061/dryad.kwh70rz7p).
