# Supplementary material for: Seasonal Habitat Distribution and Connectivity Response of Water Deer and Wild Boar to Hotspot Fencing in a Fragmented Urban Forest Fringe
Source: Ecol Evol. 2026 Mar 10;16(3):e73000. doi: 10.1002/ece3.73000 (PMC12975334; doi:10.1002/ece3.73000)
Supplement: Supplementary file 1 — Data S1: ece373000‐sup‐0001‐Supinfo.docx. [file ECE3-16-e73000-s001.docx]

**< Supplementary material S1 >**

**Journal :** Journal of Biogeography

**Title :** Seasonal Distribution and Fencing Effect for Water Deer and Wild Boar in an Urban Forest Fringe Area

**<Data Availability>**

<https://datadryad.org/stash/share/PBVhLwcEOKwOBTAEbfhc3naaShUP7XMJY6pUzSWsZTY>

Table S1 Details of camera traps at the study site.

| **No.** | **Height (m)** | **Type** | **Bearing (˚)** | **PIR sensitivity** |
| --- | --- | --- | --- | --- |
| A | 1.43 | D3 | E 90 | Normal |
| B | 1.14 | D3 | SE 129 | Normal |
| C | 1.51 | D3 | SE 132 | Normal |
| D | 1.34 | D3 | E 82 | Normal |
| E | 1.31 | D3 | S 197 | Normal |
| F | 1.43 | D3 | S 198 | Normal |
| G | 0.88 | D3 | SE 131 | Normal |
| H | 1.06 | D3 | SE 142 | Normal |
| I | 1.03 | D3 | NE 60 | Normal |
| J | 1.37 | D9 | NE 63 | Normal |
| K | 1.10 | D3 | NE 54 | Normal |
| L | 1.00 | D3 | NW 300 | Normal |
| M | 1.03 | D3 | W 258 | Normal |
| N | 1.02 | D3 | NE 38 | Normal |
| O | 0.80 | D3 | SE 150 | Normal |
| P | 1.02 | D9 | SE 150 | Normal |
| Q | 1.17 | D3 | SW 216 | Normal |
| R | 1.04 | D3 | NE 57 | Normal |

Table S2 The vegetation index used for geographical object-based image classification.

| **Vegetation index** | **Equation** | **Reference** |
| --- | --- | --- |
| G/R (GR) | G / R | Fraser et al. (2017) |
| Brightness (BI) | G+R+B | Fraser et al. (2017) |
| Normalized Greenness (Norm G) | G / (G+R+B) | Fraser et al. (2017) |
| Normalized Green-Red Ratio (Norm GR or NGRDI) | (G-R) / (G+R) | Fraser et al. (2017) |
| Excess of green (ExG) | 2G-R-B | Woebbecke et al. (1995) |

B, blue; G, green; R, red

The process of generating drone-based environmental predictor

All the layers created using drones were set at a resolution of 1 × 1 m. DEM was used to calculate slope and roughness variables in QGIS version 3.16. For distance-related variables, linear or point features (i.e., edges of forested area, sweet potato, corn, informal trails, formal trails, cemeteries, fences, mud pools, and water bodies) were extracted from the orthomosaic or identified through a field survey. After the variable extraction process, raster layers of Euclidean distances were obtained in QGIS. For roads and building variables, 1:5000 scale land use vector maps produced by the national GIS project in 2021 (source: Environmental Geographic Information Service, EGIS) were referenced to create Euclidean distances between the variables. To extract coniferous and oak trees, which provide preferred food sources for wildlife in fall and winter, the primary vegetation types were classified. This consisted of coniferous tree, oak tree, deciduous tree, and non-forest in the mountain area that were then used for geographical object-based image classification (GEOBIA) on an orthomosaic (De Luca et al. 2019). Object-based image analysis combined spectral, contextual, and morphological information of the segmentation and reduced intra-class spectral variability (Blaschke et al. 2014; Peña-Barragán et al. 2011). Utilizing improved methods of the classic pixel-oriented methods that classify pixels individually, recent research has analyzed high-resolution spatial images taken by UAVs (Modica et al. 2020; Šiljeg et al., 2022; Torres-Sánchez et al. 2015). For the GEOBIA process, segmentation and classification algorithms were implemented using the Orfeo ToolBox (OTB) within QGIS. After extracting coniferous and oak tree variables based on a random forest algorithm (Cutler et al. 2007), Euclidean distances to those variables were calculated. As a result of accuracy verification, the Kappa coefficient was 0.776 (85.6% overall accuracy). The producer’s accuracies reached 68.86% for coniferous tree and 89.04% for oak tree, and the user’s accuracies reached 83.96% for coniferous tree and 92.29% for oak tree. Appendixes 3 and 4 provide details of the GEOBIA process, including the RGB-based vegetation index, characteristics of the training and validation polygons according to the four land cover classes, and confusion matrices.

Table S3 The classification accuracy assessment with overall accuracy and Kappa coefficient.

| Classification | Reference | | | | Row total | User’s  accuracy |
| --- | --- | --- | --- | --- | --- | --- |
|  | Coniferous tree | Oak tree | Deciduous tree | Non-forest |  |  |
| Coniferous tree | 157 | 0 | 23 | 6 | 186 | 84.41% |
| Oak tree | 4 | 455 | 20 | 12 | 491 | 92.67% |
| Deciduous tree | 65 | 54 | 711 | 42 | 872 | 81.54% |
| Non-forest | 1 | 0 | 0 | 91 | 92 | 98.91% |
| Column total | 227 | 509 | 754 | 151 | 1641 | 100% |
| Producer’s  accuracy | 69.16% | 89.39% | 94.30% | 60.26% | 100% |  |
| Overall classification accuracy = 86.17% | | |  |  |  |  |
| Kappa value = 0.78 | |  |  |  |  |  |

Figure S1 Ecological response curves for Korean water deer (*Hydropotes inermis* *argyropus*). The *x*-axis represents its ranges within the study area, while the *y*-axis represents the predicted suitability of the focus variable when all of the other variables are set to their average.
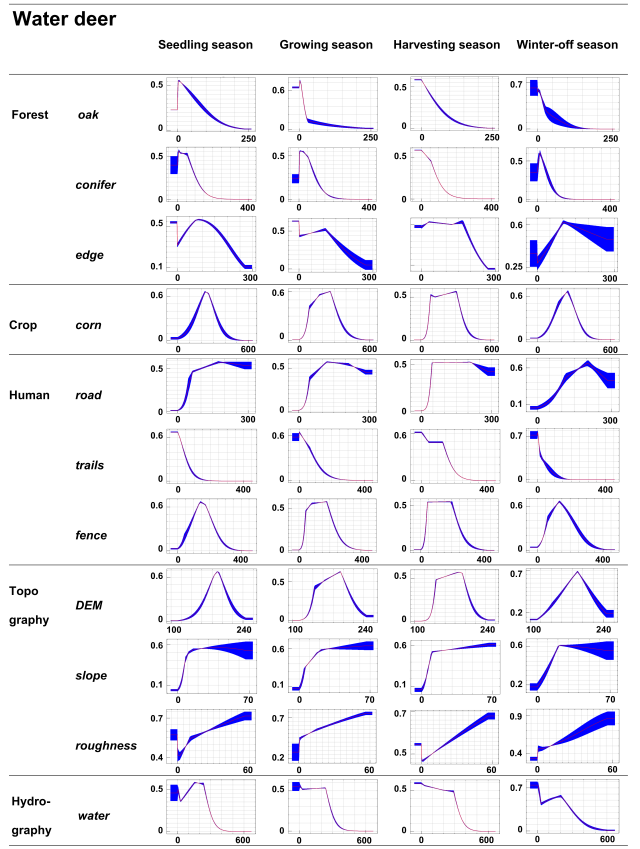


**Figure S2** Ecological response curves for wild boar (*Sus scrofa* Linnaeus). The *x*-axis of the variables represents its ranges within the study area, while the *y*-axis represents the predicted suitability of the focus variable when all of the other variables are set to their averages.


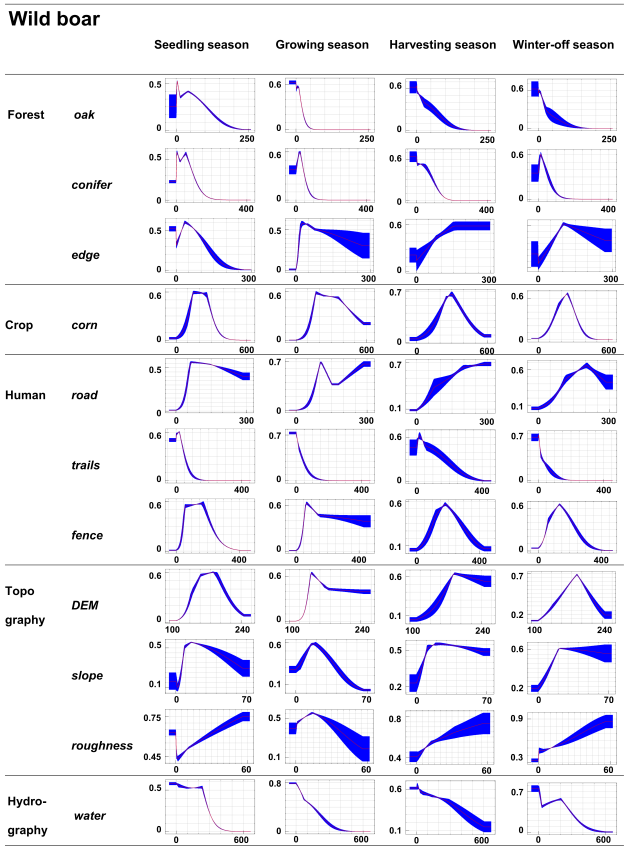


Figure S3 Predictive areas of the binary map (a) for crop rotation; during seedling season (March; 1), growing season (June; 2), harvest season (September; 3), and winter season (December; 4) at the study site. The predicted area graph for seasons (b) shows the fluctuation of the predicted presence area of water deer, wild boar, and their shared area.


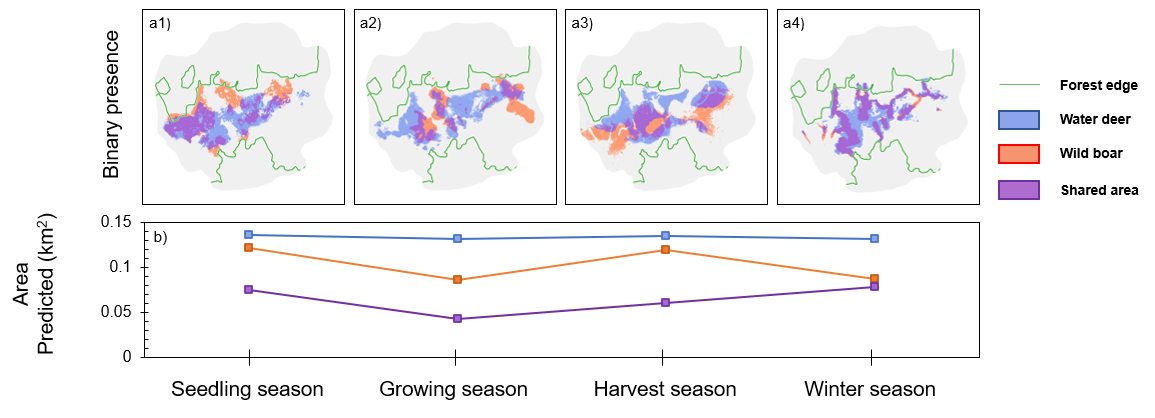


**References**

Cutler DR, Edwards TC, Beard KH, Cutler A, Hess KT, Gibson J, Lawler JJ (2007) Random forests for classification in ecology. Ecology 88:2783-2792.

Fraser RH, Van der Sluijs J, Hall RJ (2017) Calibrating satellite-based indices of burn severity from UAV-derived metrics of a burned boreal forest in NWT, Canada. Remote Sens 9:279

Modica G, Messina G, de Luca G, Fiozzo V, Praticò S (2020). Monitoring the vegetation vigor in heterogeneous citrus and olive orchards. A multiscale object-based approach to extract trees’ crowns from UAV multispectral imagery. Comput Electron Agric 175. https://doi.org/10.1016/j.compag.2020.105500

Šiljeg A, Panđa L, Domazetović F, Marić I, Gašparović M, Borisov M, Milošević R (2022) Comparative assessment of pixel and object-based approaches for mapping of olive tree crowns based on UAV multispectral imagery. Remote Sens 14. https://doi.org/10.3390/rs14030757

Torres-Sánchez J, López-Granados F, Serrano N, Arquero O, Peña JM (2015) High-throughput 3-D monitoring of agricultural-tree plantations with unmanned aerial vehicle (UAV) technology. PLoS One 10. https://doi.org/10.1371/journal.pone.0130479

Woebbecke DM, Meyer GE, Von Bargen K, Mortensen DA (1995) Color indices for weed identification under various soil, residue, and lighting conditions. Trans ASAE 38:259-269
